# Developing conductive hydrogels for biomedical applications

**DOI:** 10.1002/SMMD.20230023

**Published:** 2023-09-15

**Authors:** Yu Wang, Jiahui Guo, Xinyue Cao, Yuanjin Zhao

**Affiliations:** ^1^ Department of Rheumatology and Immunology Nanjing Drum Tower Hospital School of Biological Science and Medical Engineering Southeast University Nanjing China; ^2^ Southeast University Shenzhen Research Institute Shenzhen China

**Keywords:** biomedical applications, conductive, functionalities, hydrogels, types

## Abstract

Conductive hydrogels have attracted copious attention owing to their grateful performances, such as similarity to biological tissues, compliance, conductivity and biocompatibility. A diversity of conductive hydrogels have been developed and showed versatile potentials in biomedical applications. In this review, we highlight the recent advances in conductive hydrogels, involving the various types and functionalities of conductive hydrogels as well as their applications in biomedical fields. Furthermore, the current challenges and the reasonable outlook of conductive hydrogels are also given. It is expected that this review will provide potential guidance for the advancement of next‐generation conductive hydrogels.

## Graphical abstract

A review concerning the development of conductive hydrogels for biomedical applications has been provided. We have highlighted the recent advances in conductive hydrogels involving the various types and functionalities of conductive hydrogels as well as their applications in biomedical fields. The critical viewpoints and future improvement direction are also given. We do believe this review will provide important guidance for the future advancement of conductive hydrogels.

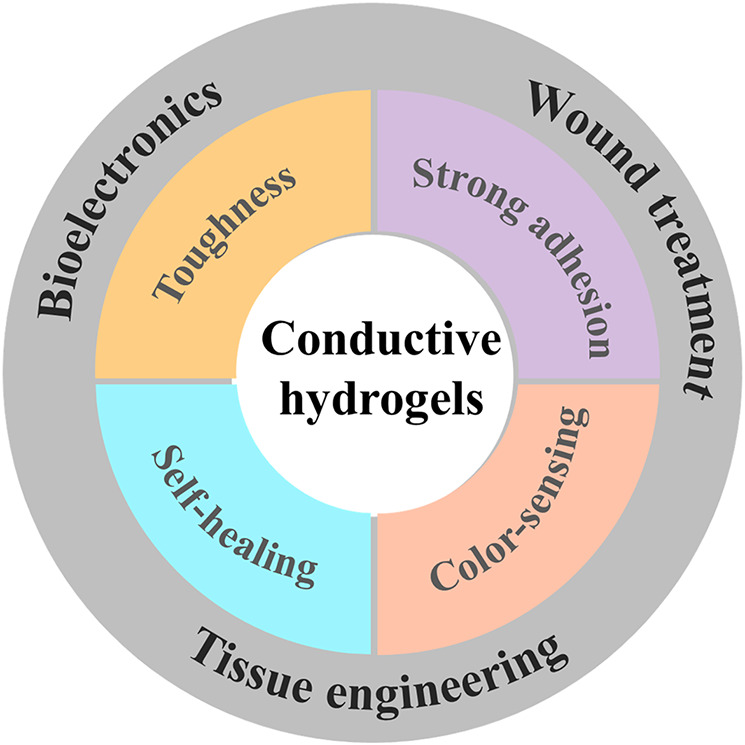

**Key points**

- Conductive hydrogels involving electronic and ionic conductive hydrogels are introduced.
- The functionalities of conductive hydrogels as well as their typical biomedical applications are discussed.
- Current challenges and future research directions of conductive hydrogels have been prospected.

## 1 INTRODUCTION

Conductive hydrogels, mainly composed of polymeric matrix and conductive medium, have aroused tremendous interests for their unique features, such as comparable tissue‐like mechanical performance, intrinsic electrical conductivity and biocompatibility.^1^, ^2^, ^3^, ^4^, ^5^, ^6^ Over the last decades, various types of conductive hydrogels have been developed, which can intelligently respond to biological tissues through electricity, showing great potential in the biomedical field.^7^, ^8^, ^9^, ^10^, ^11^, ^12^ Generally, these conductive hydrogels can be classified into two genres, including electronic, and ionic conductive hydrogels.^13^, ^14^, ^15^, ^16^, ^17^, ^18^ Although these achievements suffer from harsh physiological environments and complex biomedical applications, conventional conductive hydrogels cannot meet the requirements of specific practical applications.^19^, ^20^, ^21^, ^22^, ^23^, ^24^ It is urgent for conductive hydrogels to improve their performance regarding conductive sensitivity, mechanical stability, durability as well as biocompatibility, thus imparting them with sufficient capability to interact with biological systems. Fortunately, continuous efforts have been made to the development of conductive hydrogels with multifunctionality, such as stretchability, strong adhesion, self‐healing, and color‐sensing, which have greatly promoted their performance and further broadened their potentials in biomedical applications.^25^, ^26^, ^27^, ^28^, ^29^, ^30^, ^31^

Herein, we present the latest advancements in conductive hydrogels for biomedical applications. To begin with, various conductive hydrogels have been summarized in terms of electronic and ionic conductive hydrogels. Secondly, their functionalities including toughness, strong adhesion, self‐healing and color‐sensing are discussed. Next, their typical biomedical applications are described including bioelectronics, wound treatment and tissue engineering. Finally, we made a brief conclusion and provided our glimpse of challenges and future outlook for the next generation of conductive hydrogels.

## 2 TYPES OF CONDUCTIVE HYDROGELS

As composed of conductive fillers such as conductive polymers, metallic particles and carbon‐based materials, the conductive hydrogels are endowed with electronic conduction.^32^ Another representative conductive hydrogels are obtained by incorporating free ions such as charged salts and ionic liquids, which are defined as ionic conduction.^33^ In this section, we will introduce the two types of conductive hydrogels regarding their design and features.

### 2.1 Conductive hydrogels based on electronic conduction

#### 2.1.1 Conductive polymers‐based conductive hydrogels

Conductive polymers are considered as a class of organic materials, which can undergo electron transfer along the whole backbone.^34^, ^35^, ^36^ Several conductive polymers are commonly used for the construction of conductive hydrogels, including poly(3,4‐ethylenedioxythiophene) polystyrene sulfonate (PEDOT:PSS), polyacetylene, polyaniline (PANi), polythiophene, polypyrrole (Ppy), etc.^37^, ^38^, ^39^, ^40^, ^41^ One of the universal approaches of conductive polymers for the fabrication of conductive hydrogels is to blend with cross‐linked hydrogel networks.^42^ Although great achievements have been made, conductive hydrogels relied on conductive polymers still have the shortcomings of mechanical brittleness and water insolubility resulting from the stiffness of conjugated polymer chains and hydrophobic networks, which dramatically decreases the performances of conductive hydrogels. Adopting dopants, such as dopamine (DA), phytic acid (PA), tannic acid (TA) or polyelectrolytes, has been proven to be a useful method to solve these problems.^43^, ^44^, ^45^ Zhang et al. proposed a conductive hydrogel taking advantage of polydopamine@Ppy (PDA@Ppy) nanocomposite which was prepared by the copolymerization of DA and pyrrole.^46^ The addition of PDA not only overcame the hydrophobicity of Ppy to avoid the aggregation phenomenon but also provided more reaction sites, further improving the cross‐linked degree of the resultant conductive hydrogels.

**FIGURE 1:**
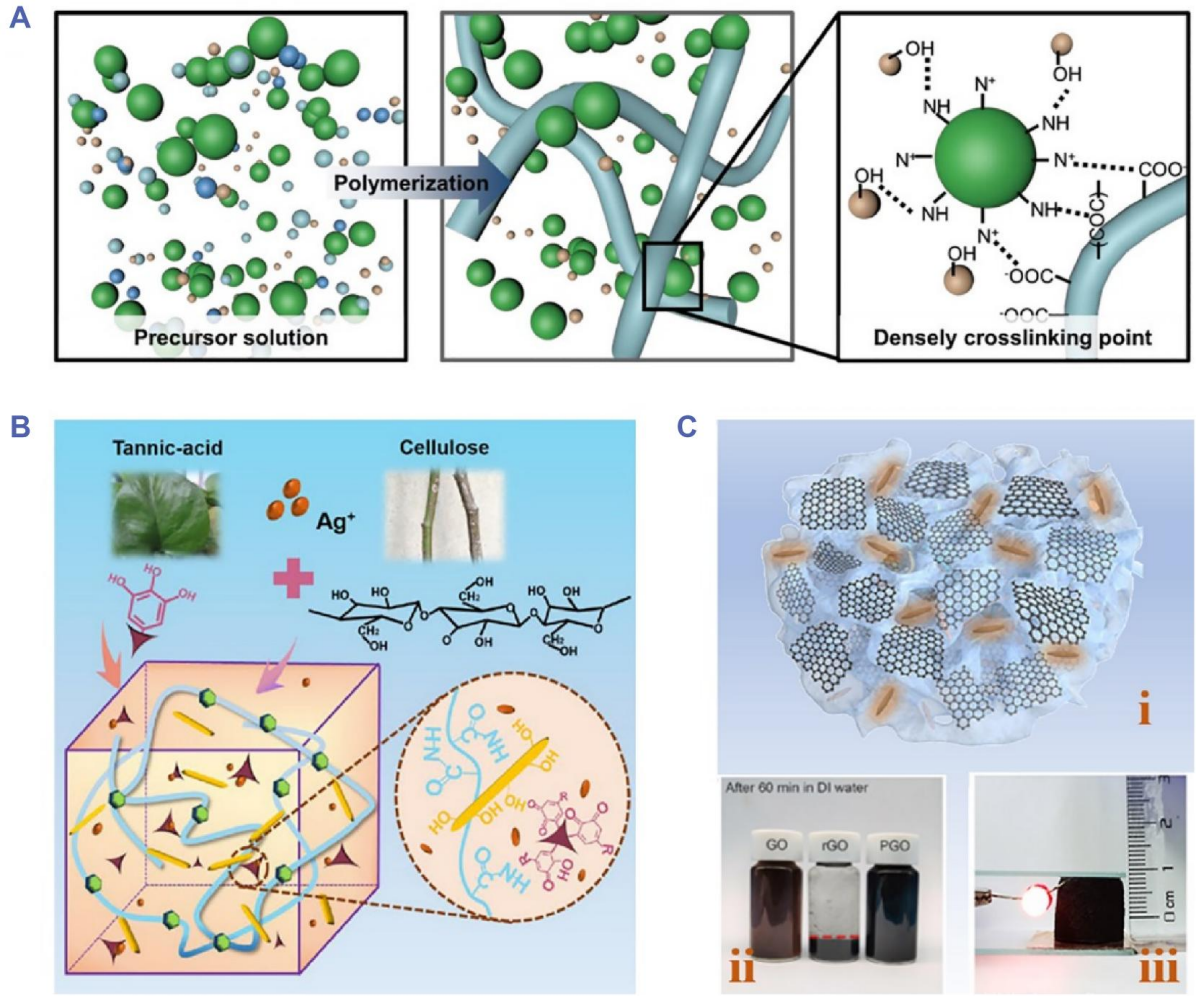
Electronic conductive hydrogels. (A) Schematic diagram of the PANi‐NPs‐based conductive hydrogel. *Source*: Reproduced with permission.^48^ Copyright 2022, John Wiley and Sons. (B) Schematic illustration of the free‐radical polymerization of PAM/TA‐Ag/cellulose nanocrystals conductive hydrogel. *Source*: Reproduced with permission.^49^ Copyright 2020, American Chemical Society. (C) The fabrication of PGO‐PHA‐AG conductive hydrogel. (i) The scheme of PGO‐PHA‐AG conductive hydrogel scaffold. (ii) Digital image of GO, rGO and PGO dispersed in water for 1h. (iii) Digital image of PGO‐PHA‐AG conductive hydrogel scaffold lighting a bulb. *Source*: Reproduced with permission.^50^ Copyright 2022, The Authors, published by Elsevier.

Additionally, in situ polymerization of conductive monomers and hydrogel precursor is another effective strategy to protect conductive polymers from inadequate dispersibility.^47^ For example, a conductive hydrogel was reported by introducing acrylic acid (AA), poly (ethylene glycol) methacrylate (PEG), PA and aniline (ANi) (Figure 1A).^48^ Based on a multiphase synthesis technique, PANi‐nanoparticles (PANi‐NPs) were obtained and uniformly dispersed in the polymer monomer solution. Besides, intermolecular interactions appeared, which greatly enhanced interfacial interactions. After the following free radical polymerization, the conductive hydrogel was acquired, which showed improved electronic conductivity (74.32 mS·cm^−1^). Moreover, to deal with the mechanical brittle nature of conductive polymers, double networks (DN) can be introduced into conductive hydrogels, which is achieved by adopting a non‐conductive matrix as the first supporting network and the conductive polymer as the second network.^51^ The interpenetration networks can impart the conductive hydrogels with greater mechanical performance.

#### 2.1.2 Metallic particles‐based conductive hydrogels

Benefiting from excellent conductivity, mechanical flexibility and high surface energy, many metals (gold (Au)/silver (Ag)/copper (Cu)) have been explored and showed distinctive performance for the design of conductive hydrogels.^52^ Although with these merits, metals show weak interfacial interaction with hydrogels due to poor compatibility. To settle this issue, the modification of metals has been demonstrated to be an effective method.^53^ Specifically, incorporating hydrophilic materials or compounds with reducing ability is beneficial to in situ obtain and stabilize metallic nanomaterials, further contributing to realizing conductive hydrogels. Yang et al. adopted TA to reduce Ag^+^ to AgNPs, which induced the reconversion of quinones to catechol groups, further favoring free‐radical polymerization of cellulose nanocrystals (Figure 1B).^49^ The resultant polyacrylamide (PAM) conductive hydrogel showed great mechanical performance based on the formation of hydrogen bonds between cellulose nanocrystals and TA‐Ag complexes.

#### 2.1.3 Carbon‐based materials‐based conductive hydrogels

Carbon‐based materials own the advantages of remarkable electrical and optical performances, stable mechanical strength as well as excellent flexibility, which are potential conductive fillers for conductive hydrogels. Carbon nanotubes (CNTs), graphene (GO) and its derivatives are typical carbon‐based materials.^54^ The method for designing conductive hydrogels involves introducing conductive fillers into the hydrogel network. Owing to the adjustable addition of conductive fillers, the conductivity of carbon‐based materials‐based conductive hydrogels is tunable; meanwhile, the amount change of conductive fillers can influence the interactions with hydrogels, further controlling the mechanical properties of conductive hydrogels. Despite the flexibility and controllability, an inevitable issue remains regarding severe aggregation, which hinders the applications of carbon‐based materials. To address this issue, carbon‐based materials can be modified by hydrophilic polymers, thus improving their compatibility with hydrogels. For instance, a conductive alginate/gelatin (AG) scaffold was developed consisting of PDA‐modified GO (PGO) and PDA‐modified hydroxyapatite nanoparticles (PHA) (Figure 1C).^50^ The modification of PDA contributed to improve both dispersibility and conductivity, which resulted in the partial reduction of GO.

### 2.2 Ionic conductive hydrogels

Different from electronic conductive hydrogels, ionic conductive hydrogels transport electrical signals through free moving ions, which can be realized through three‐dimensional network structured hydrogels with multichannels for ion migration. Free ions can be obtained from polyelectrolyte hydrogels, electrolytes or ionic liquids.^55^ Owing to the unique compositions and conductive mechanism, ionic conductive hydrogels exhibit transparency morphology, tunable mechanical property and stable conductivity even suffering from large deformations.

#### 2.2.1 Electrolyte‐based ionic conductive hydrogels

**FIGURE 2:**
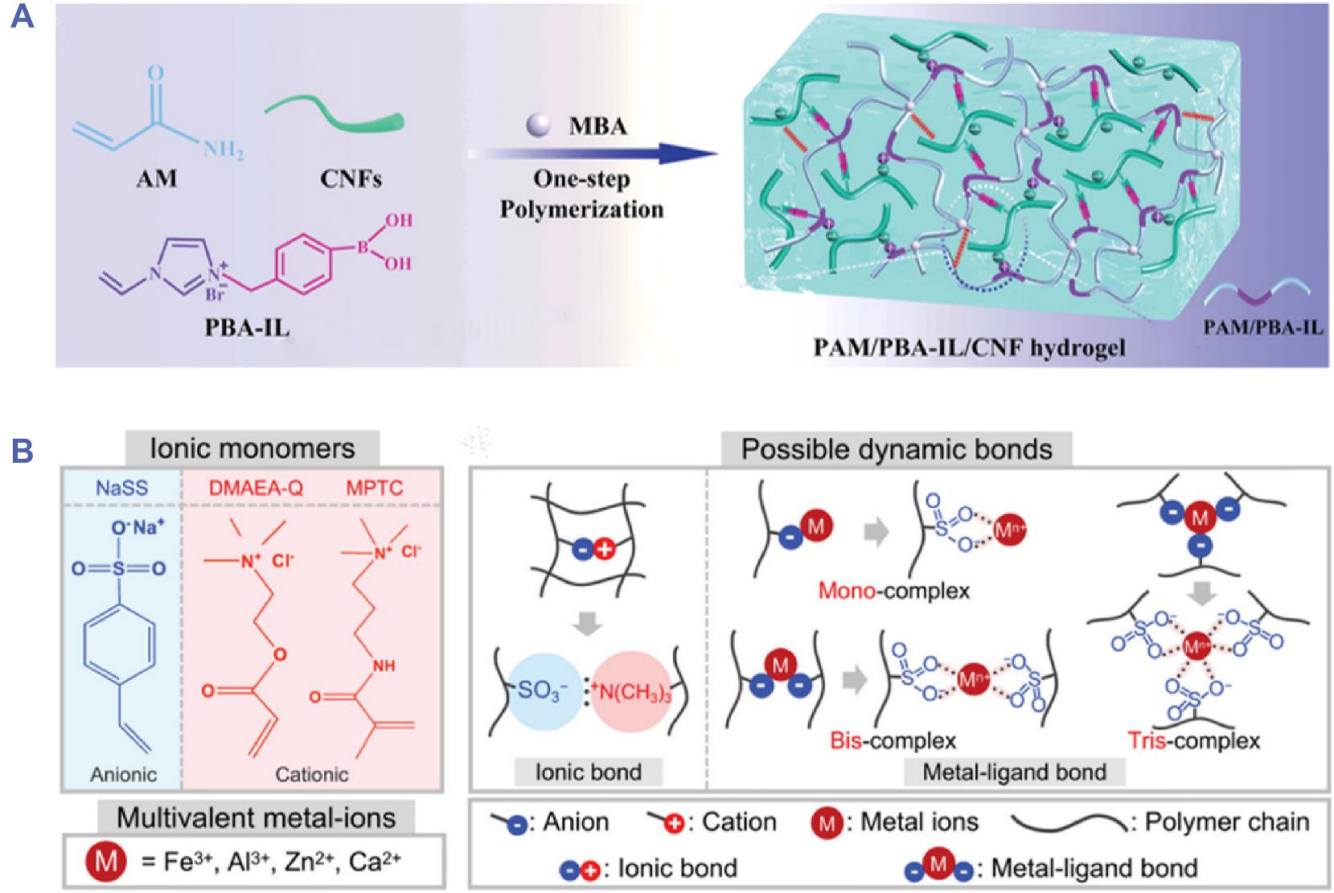
Ionic conductive hydrogels. (A) Schematic illustration of the fabrication process of PAM/PBA‐IL/CNF electrolyte‐based ionic conductive hydrogels. *Source*: Reproduced with permission.^57^ Copyright 2022, John Wiley and Sons. (B) Chemical structure of ionic monomers, multivalent metal ions and possible dynamic bonds in polyampholyte‐based ionic conductive hydrogel. *Source*: Reproduced with permission.^58^ Copyright 2021, John Wiley and Sons.

Generally, two approaches are widely applied for the construction of electrolyte‐based ionic conductive hydrogels, involving directly mixing conductive ion solution with hydrogel precursor and immersing the as‐prepared hydrogels in conductive ion solution.^56^ Ionic liquids, a kind of organic salt solvent with low melting points, show unique physical and chemical properties, including remarkable conductivity, low vapor pressures, high thermal and chemical stability, etc. Benefitting from these features, ionic liquids have been regarded as versatile electrolytes for the construction of conductive hydrogels. In recent studies, Han and colleagues fabricated a high‐performance ionic conductive hydrogel, which used phenylboronic acid‐ionic liquid (PBA‐IL) as an ionic conductor and PAM as the hydrogel network (Figure 2A).^57^ By mixing them and taking advantage of cellulose nanofibrils (CNFs) to enhance dynamic crosslinking networks, the ionic conductive hydrogel could achieve superior stretchability (1810 ± 38%), high transparency and sensitive conductivity (Gauge factor = 8.36). In addition, free inorganic acids, alkalis and salts can be introduced into hydrogel networks to provide free ions, such as NaCl, KCl, LiCl, boric acid, etc. An ionic conductive hydrogel was presented by immersing carboxymethyl chitosan (CS) hydrogel in LiCl aqueous solution, in which LiCl could be soaked through osmotic pressure difference.^59^ The conductivity of the ionic hydrogel was dependent on the salt ion concentration; meanwhile, the participation of salt ions could impart the resultant ionic hydrogel with anti‐drying property.

#### 2.2.2 Polyelectrolyte‐based ionic conductive hydrogels

Different from electrolyte‐based ionic conductive hydrogels, polyelectrolyte‐based ionic conductive hydrogels can be fabricated using polyelectrolyte hydrogels, which are obtained through ionic bonds from the reaction between the cationic and anionic monomer.^60^ The hydrogel network with unique mesh structure can provide sufficient space for the migration of water molecules and ions, and ion channels can be formed using polyelectrolytes, thus achieving good conductivity. Common polyelectrolytes include alginate, CS, hyaluronic acid, polyacrylic acid (PAA), polymethacrylic acid (PMAA), and et al.^61^, ^62^ Besides, polyampholyte hydrogels, which possess anionic and cationic groups, are also widely used for the construction of ionic conductive hydrogels. In recent work, one polyampholyte hydrogel by copolymerization of anionic sodium p‐styrenesulfonate (NaSS) and cationic dimethylaminoethylacrylate quaternized ammonium (DMAEA‐Q) was proposed.^58^ Although the dynamic ionic network could be formed through the polyampholyte hydrogel, poor mechanical performance remained. To solve this problem, metals could be introduced into polyampholyte hydrogels to form metal‐ligand bonds so that good mechanical strength was achieved. It was demonstrated that multivalent metal‐ion (Fe^3+^) was introduced into the dynamic ionic network, which dramatically improved the mechanical properties in terms of Young's modulus, tensile fracture strength and tension (Figure 2B).

In comparison with electronic conductive hydrogels, ionic conductive hydrogels show higher mechanical performance and more transparency. However, ionic conductive hydrogels also have the limitations that the balance of ion concentration and water content should be controlled well, which will hinder their conductivity. Moreover, changes in temperature and humidity would affect the conductivity of ionic conductive hydrogels as well.

## 3 FUNCTIONALITIES OF CONDUCTIVE HYDROGEL

To impart conductive hydrogels with practical value, diverse functionalities such as toughness, strong adhesion, self‐healing and color‐sensing have been investigated and integrated into conductive hydrogels. In this section, we will introduce them and discuss their roles in improving the performances of conductive hydrogels.

### 3.1 Toughness

Mostly, conductive hydrogels are limited by poor mechanical performance in terms of mechanical and tensile strength, which seriously weakens their matching with biological tissues and further restricts their biomedical applications. Various strategies have been proposed to settle the shortcomings, such as the introduction of DNs, the addition of nanomaterials, and adopting interpenetrating polymer networks.^63^, ^64^, ^65^ The critical principle of these approaches to improve toughness involves an effective dissipation mechanism when restoring to the original state after suffering from large deformations and undamaged configurations.

**FIGURE 3:**
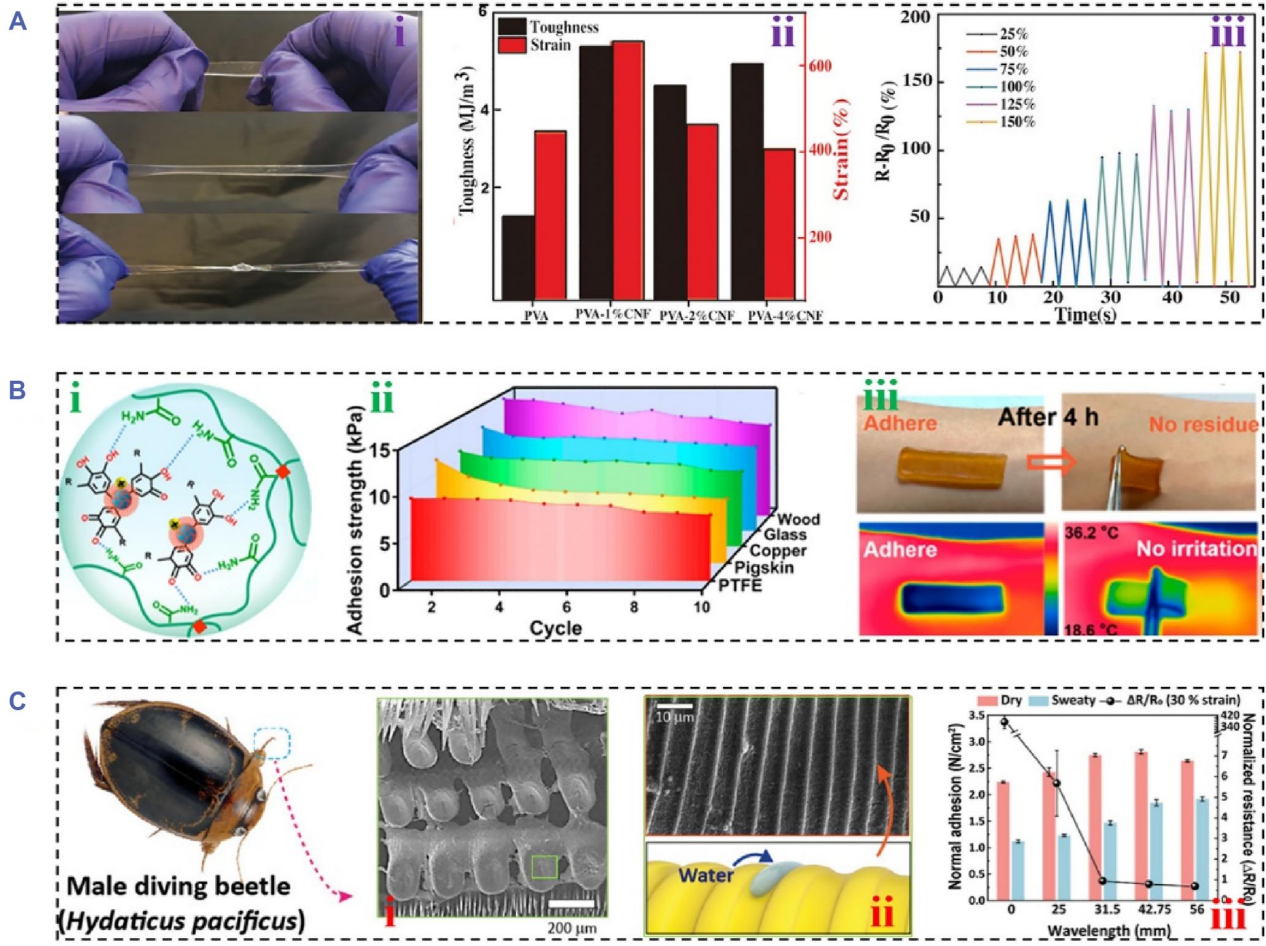
Toughness and strong adhesion performance. (A) (i) Digital images of PVA/CNFs conductive organohydrogel under stretching. (ii) Toughness and strain property of PVA/CNFs conductive organohydrogel. (iii) Relative resistance changes with the variations of stains. *Source*: Reproduced with permission.^67^ Copyright 2020, John Wiley and Sons. (B) The mechanism diagram of LSN‐Fe/PAM conductive hydrogels (i). The adhesion ability of LSN‐Fe/PAM hydrogels adhered on different substrates (ii). The photographs of LSN‐Fe/PAM hydrogels adhered on the skin (iii). *Source*: Reproduced with permission.^68^ Copyright 2022, American Chemical Society. (C) Photographs of male diving beetles and their foreleg microstructures (i). The enlarged view of microstructures of male diving beetle's forelegs and schematic diagram of the wrinkled channels for drainability (ii). The relationship of relative resistance changes and adhesion strength with 30% strain (iii). *Source*: Reproduced with permission.^69^ Copyright 2022, John Wiley and Sons.

Taking inspiration from this principle, researchers have presented many conductive hydrogels with desirable toughness and mechanical strength. For instance, DNs based on double‐strand κ‐carrageenan (κ‐CG) helices and poly(acrylamide ‐co‐acrylic acid) (P(AAm‐co‐AAc)) were constructed for conductive hydrogels.^66^ Meanwhile, the metal‐ion coordination bond (Fe^3+^) was crosslinked with P(AAm‐co‐AAc) to further enhance the mechanical strength. Such a triple noncovalent crosslinking mechanism was responsible for outstanding tensile strength (2.7 MPa), strong fracture strain (1400%) and brilliant toughness (9.82 MJ m^−3^). In addition, Jiang et al. developed a highly stretchable ionic organohydrogel by endowing polyvinyl alcohol (PVA) as the primary polymer network and CNFs as reinforcement nanofiller (Figure 3A).^67^ Benefitting from intermolecular hydrogen bonding between polymer chains and CNFs, the resultant conductive hydrogels showed excellent toughness regarding strong elongation (up to 660%) (Figure 3Ai), tensile strength (2.1 MPa) and toughness (5.25 MJ m^−3^) (Figure 3Aii). The brilliant toughness made conductive hydrogels show stable conductive transmission even suffering from stretching up to 150% strain (Figure 3Aiii).

### 3.2 Strong adhesion

Due to the wet dynamic physiological environment involving physiological fluid or blood, strong adhesion is important for conductive hydrogels when applied in biomedical fields. Notably, strong adhesion not only improves comfortable contact with tissues but also facilitates stable electrical signal transmission.^70^, ^71^ However, existing conductive hydrogels show poor adhesion, and there remain challenges in achieving strong adhesion ability. To resolve the issue, efforts have concentrated on the introduction of dynamic bonds. Yang's group fabricated a flexible conductive hydrogel with self‐adhesion capability by a dynamic redox reaction in sulfonated lignin‐coated silica nanoparticles (LSNs)‐Fe/PAM hydrogels (Figure 3B).^68^ Dynamic and reversible hydrogen bonds could be formed between LSNs and FeCl_3_ complex, which resulted in highly adhesive strength through energy dissipation (Figure 3Bi). The reported hydrogel exhibited the ability of long‐lasting strong adhesion of different substrates after 10 cycles and robust self‐adhesion (14 kPa adhesion strength to wood) (Figure 3Bii). Besides, seamless adhesion to human skin was demonstrated (Figure 3Biii).

Apart from dynamic bonds, biomimetic organisms' microstructures, topological entanglement of polymer chains and integration of extra adhesives are commonly methods to impart conductive hydrogels with strong adhesion property.^72^, ^73^, ^74^ Taking inspiration from the wrinkled suction cups of a male diving beetle, Pang and colleagues fabricated an adhesive conductive polydimethylsiloxane (PDMS) film implanted with CNTs (Figure 3Ci).^69^ Such unique bioinspired architecture with mushroom‐shaped pillars was constructed by the replicating molding and selective‐transfer method, which could be generated by a negative pressure under water, endowing it with great adhesion. In particular, microwrinkles were detected on the surface of the pillars, which could serve as wrinkled channels for drainability (Figure 3Cii). Benefitting from the bio‐inspired structures, the resultant film was imparted with water‐repellent adhesion, and the adhesive strength could reach up to 1.5 N cm^−2^ on sweaty pig skin (Figure 3Ciii). In contrast, Liu et al. incorporated topologically entangled PDA into a PAM network, which was locked by metal coordination bonds to realize a self‐adhesive conductive hydrogel.^75^ Besides, to avoid insensitive data acquisition, the introduction of individual adhesive layer was demonstrated as a useful method for building adhesive conductive hydrogels. A sandwiched structure consisting of an elastic layer (PVA/PAM), a conductive layer (CNTs/GO) and an adhesive layer (PAA/glycerol) was constructed.^76^ The adhesive layer not only made the conductive hydrogel adhere on various surfaces, such as plastic, glass, ceramics, steel, and human skin, but also favored to detect tiny strain.

### 3.3 Self‐healing

For biomedical applications, there is an essential requirement to construct conductive hydrogels with self‐healing capability, which can greatly improve their resistance to inevitable mechanical damage in practical use and extend service life. Until now, many strategies have been proposed and the general principles can be divided into the dynamic covalent cross‐linking (Diels‐Alder reaction, borate ester bonds, Schiff bases, etc.) and physical interactions (metal coordination bonds, hydrogen bonds, ionic bonds, etc.).^77^, ^78^, ^79^, ^80^ Dynamic covalent cross‐linking is a kind of static chemical cross‐linking involving permanent bonds, which can be broken and reformed. In recent studies, Gu's group developed a self‐healing DN conductive hydrogel by using CS with dialdehyde carboxymethyl cellulose (DCMC) as the first network and PAA with Al^3+^ as the second network.^81^ Schiff base bonds were formed between CS and DCMC, which endowed the hydrogels with superior underwater self‐healing performance with 51.7% self‐healing rate, indicating their potential in complex environments.

**FIGURE 4:**
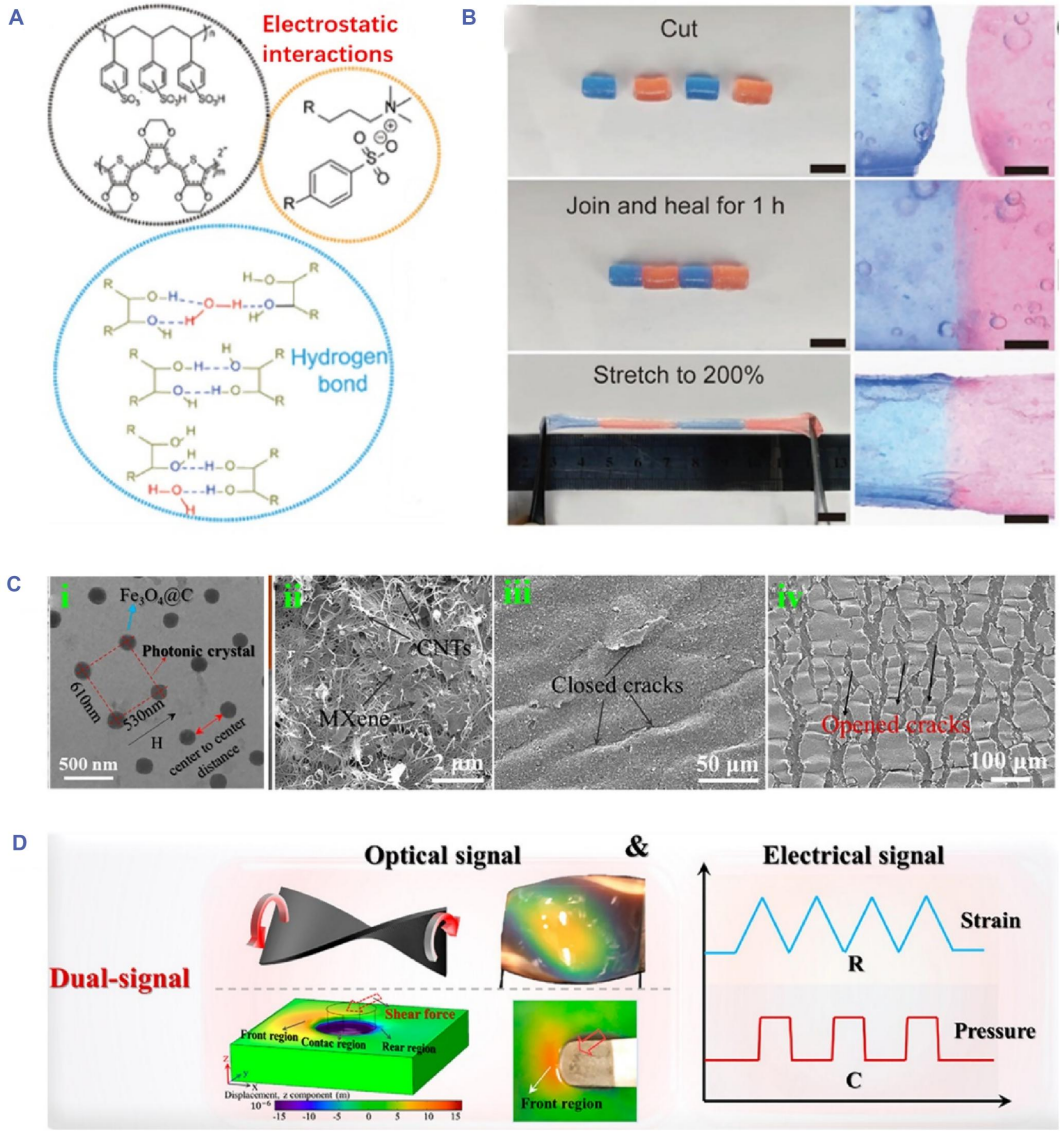
Self‐healing and color‐sensing properties. (A) Schematic illustration of electrostatic interactions and hydrogen bonding of the injectable conductive hydrogel. (B) Photographs of the self‐healing process of broken injectable conductive hydrogel blocks. (A, B) *Source*: Reproduced with permission.^82^ Copyright 2020, John Wiley and Sons. (C) Transmission electron microscope (TEM) images of Fe_3_O_4_@C (i), Scanning electron microscope (SEM) images of CNT/MXene film (ii), closed microcracks (iii) and opened microcracks (iv). (D) Schematic diagram of optical/electrical skin with dual‐signal response properties. (C, D) *Source*: Reproduced with permission.^83^ Copyright 2023, American Chemical Society.

Compared with dynamic covalent cross‐linking, physical interactions have features of weaker bond strength, good reversibility and easy preparation. Jiang et al. fabricated an injectable conductive hydrogel with self‐healing ability through hydrogen bonding and electrostatic interactions (Figure 4A).^82^ Specifically, the hydration of cationic guar gum (CG) was electrostatic interactions with PEDOT:PSS. Followed by alkali neutralization and increased hydrogen bonding among chains, the conductive hydrogels (PPGS) could be formed within 1 min. It was demonstrated that the obtained hydrogels showed excellent deformability and great self‐healing property, which could be deformed as desired, and self‐healed within 30 min without extra treatment attributed to the formation of dynamic hydrogen bonding in the gel (Figure 4B).

### 3.4 Color‐sensing

Considering complex biological environments, electrical signals may be interfered, and a single signal input or output has difficulty in reflecting the condition of the object. Structural color has emerged and can be integrated into conductive hydrogels for the improvement of the accuracy and stability of signal acquisition.^84^ Structural color is a kind of photonic material caused by Bragg reflection of light in a periodic arrangement structure. Based on the structural color phenomenon, various structural color materials have been developed with color‐sensing performances, which have been widely applied in sensors, anticounterfeiting and displays.^85^, ^86^, ^87^, ^88^ Such color‐sensing property is beneficial for the construction of dual‐signal conductive hydrogels, which can obtain both electronic and optical signal feedback in response to external stimuli, further improving signal reliability.

The periodic alignment of colloidal crystals imparts them with structural color, which has been employed as the template for the fabrication of inverse opal structured hydrogels.^89^, ^90^, ^91^, ^92^ An ionic conductive hydrogel with structural color property was developed by our group from the inverse opal structure.^93^ Such inverse opal scaffold derived from silica nanoparticles (SiO_2_) exhibited that the vivid structural color and the conductivity were due to the migration of lithium ions (Li^2+^). Once experiencing external stimuli such as stretching or pressing, an evident structural color variation appeared. Notably, the dual‐signal sensing could be achieved through visual monitoring and real‐time electrical feedback, demonstrating practical value in monitoring human activity. Apart from SiO_2_, it is worth noting that the non‐close‐packed Fe_3_O_4_ nanoparticles can also generate structural color with mechanochromic property. Kim et al. proposed an optical/electrical skin by adopting Fe_3_O_4_ photonic crystals as the optical sensing element and CNT/MXene with a microcrack structure as the hybrid conductive element (Figure 4C).^83^ It was seen that the embedded Fe_3_O_4_@C nanoparticles showed a large center‐to‐center distance (Figure 4Ci). Such a non‐closed‐packed structure endowed the film with vivid structural color. The great conductivity was derived from the uniform distribution of CNTs and MXene (Figure 4Cii). Once suffering from stretching, microcracks were formed, imparting the film with ultrasensitive capability (Figure 4Ciii‐iv). The proposed film could realize strain and pressure sensing by accurate color‐switching optical signal and ultrasensitive conductivity signals originating from the microcrack structure (Figure 4D). Such dual‐signal response combining conductive hydrogels and structural color may provide deep insights into the advancements of conductive hydrogels and broaden their applications.

## 4 BIOMEDICAL APPLICATIONS

Owing to their advantages such as comparable tissue‐like mechanical performance, intrinsic electrical conductivity and biocompatibility as well as advanced functionality including toughness, adhesion, self‐healing and color‐sensing, conductive hydrogels are expected to provide remarkable values in biomedical applications. They have been employed for bioelectronics, controllable drug release, wound treatment, and tissue engineering, which are presented in detail in this section.

### 4.1 Bioelectronics

Bioelectronics is a subject of combination of biology system and electronics, which can transduce electrophysiological or physical signals into electrical signals, contributing to disease prevention and diagnostics.^94^ Great achievements have been obtained in bioelectronics, among which tremendous attention has been concentrated on wearable bioelectronics with the advantages of electrical response to different degrees of deformation. Conductive hydrogels show great potential in wearable electronics, which can be devoted to personalized health monitoring and management through capturing valuable information and health status from the body in terms of a variety of physiological signals, such as human motions, heart or pulse rate, etc.^95^, ^96^, ^97^

**FIGURE 5:**
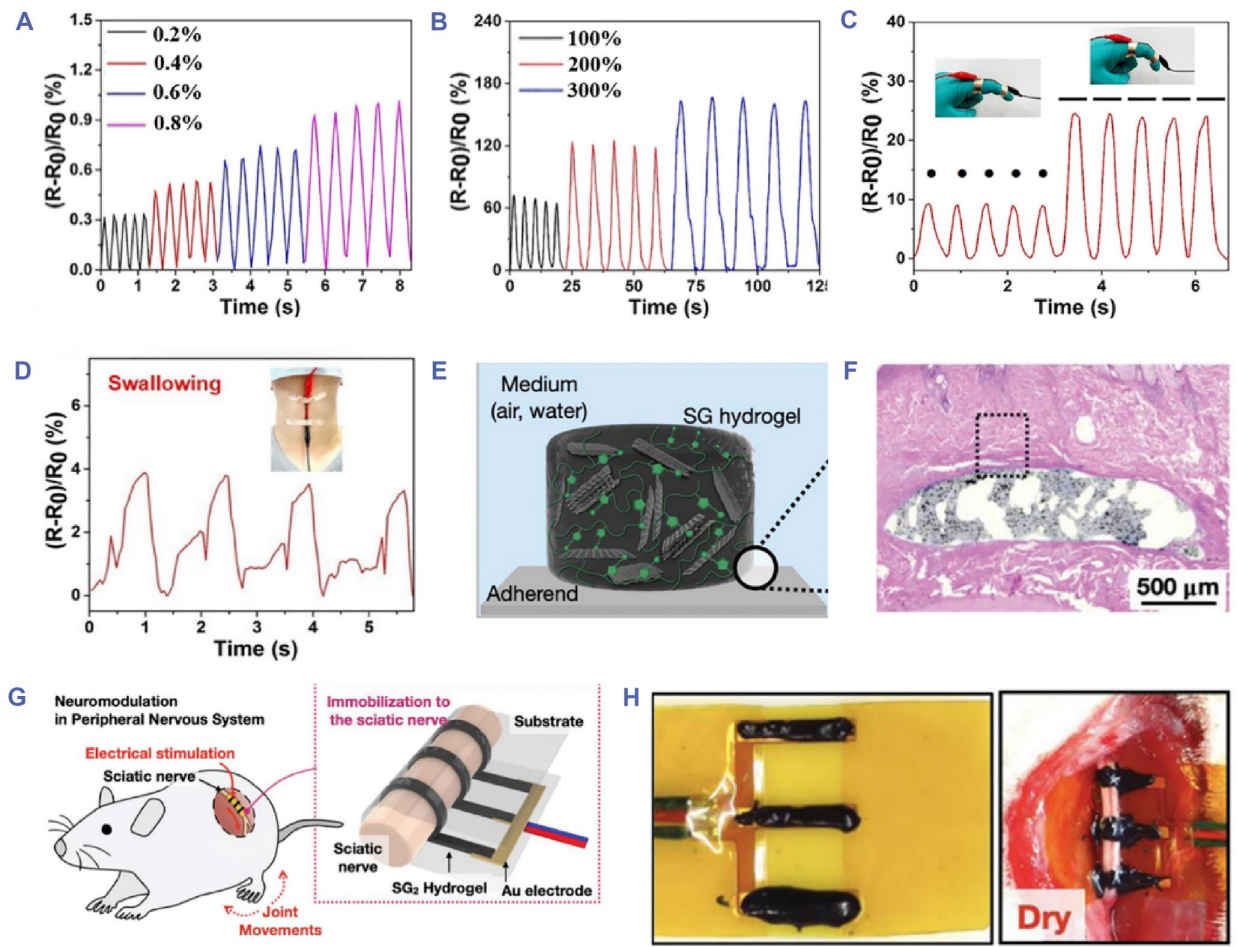
Bioelectronic applications. (A, B) Relative resistance changes facing with small strain (A) and large strain (B). (C, D) Relative resistance changes when monitoring human finger motions (C) and swallowing (D). (A‐D) *Source*: Reproduced with permission.^98^ Copyright 2022, The Royal Society of Chemistry. (E) Schematic diagram of SG hydrogel. (F) H&E staining image of hydrogel‐tissue interface. (G) Schematic illustration of the SG hydrogel applied to the peripheral nervous system. (H) Images of the SG hydrogel adhered to the sciatic nerve. (E‐H) *Source*: Reproduced with permission.^99^ Copyright 2023, John Wiley and Sons.

Recently, Sun’s group presented a conductive hydrogel based on DN through combining a hydrophobic‐conjugated hydrogel and an ionic coordination network.^98^ The proposed conductive hydrogels exhibited superior toughness (60 MJ/m^3^) and great sensitivity, showing practical value in serving as a strain sensor. It was demonstrated that the conductive hydrogel could generate electrical response to both slight strain (0.2%–7%) and large strain (10%–300%) (Figure 5A–B). Such sensitive responsive property to different ranges of deformations was conducive to monitoring human activities from large‐scale joint motions to slight swallowing or light coughing (Figure 5C–D). The results identified the role of conductive hydrogels for human motion monitoring, which would be superior valuable in personal medical diagnostics. In addition, a liquid metal‐based conductive film was designed applied for a stress sensor to distinguish the pronunciation of different letters through electrical signal variations induced by vibrations.^100^ Besides, Mecerreyes's group presented a wearable sensor derived from gelatin‐based PEDOT: lignin sulfonate eutectogels.^101^ Good adhesion favored conformal contact with the skin, and the epidermal physiological signals including electrocardiogram (ECG) and electromyogram (EMG) could be acquired by attaching the film to the wrist. Notably, real‐time and long‐term monitoring could be achieved, which made a great contribution to human health management.

Besides in vitro wearable bioelectronics, in vivo bioelectronics can be achieved by implanting conductive hydrogels into target tissues. The mechanical properties of implanted conductive hydrogels should be matched with target tissues with foreign‐body reactions avoided. Meanwhile, good adhesion is essential for implanted applications, which can enhance the interfacial interactions between materials and soft tissues.^102^, ^103^, ^104^ However, conductive hydrogels have limitations of swelling in the moist physiological environment, which dramatically restrict their mechanical property as well as adhesion ability, and eventually have a negative influence on their conductivity. To solve the problem, Kim et al. proposed a non‐swelling graphene‐based poly(sulfobetaine vinylimidazolium) conductive hydrogel (SG hydrogel) and investigated its potential in implanted bioelectronics (Figure 5E).^99^ It was demonstrated that the conductive hydrogel showed suitable viscoelasticity to tissues, non‐swelling ability, good adhesion to living tissues, and great conductivity (4.9 × 10^−5^ S cm^−1^). Notably, the conductive hydrogel could be subcutaneously implanted for 3 weeks, and hematoxylin and eosin (HE) staining was observed the reduction of inflammatory expression, exhibiting good biocompatibility (Figure 5F). The remarkable performances made it an ideal implant for neuromodulation in the peripheral nervous system (Figure 5G). The results demonstrated that the conductive hydrogel could be implanted stably even in a wet environment, and effective electrical stimulation could be applied to force the rat leg movement, confirming its potential for next‐generation bioelectronics (Figure 5H).

### 4.2 Wound treatment

**FIGURE 6:**
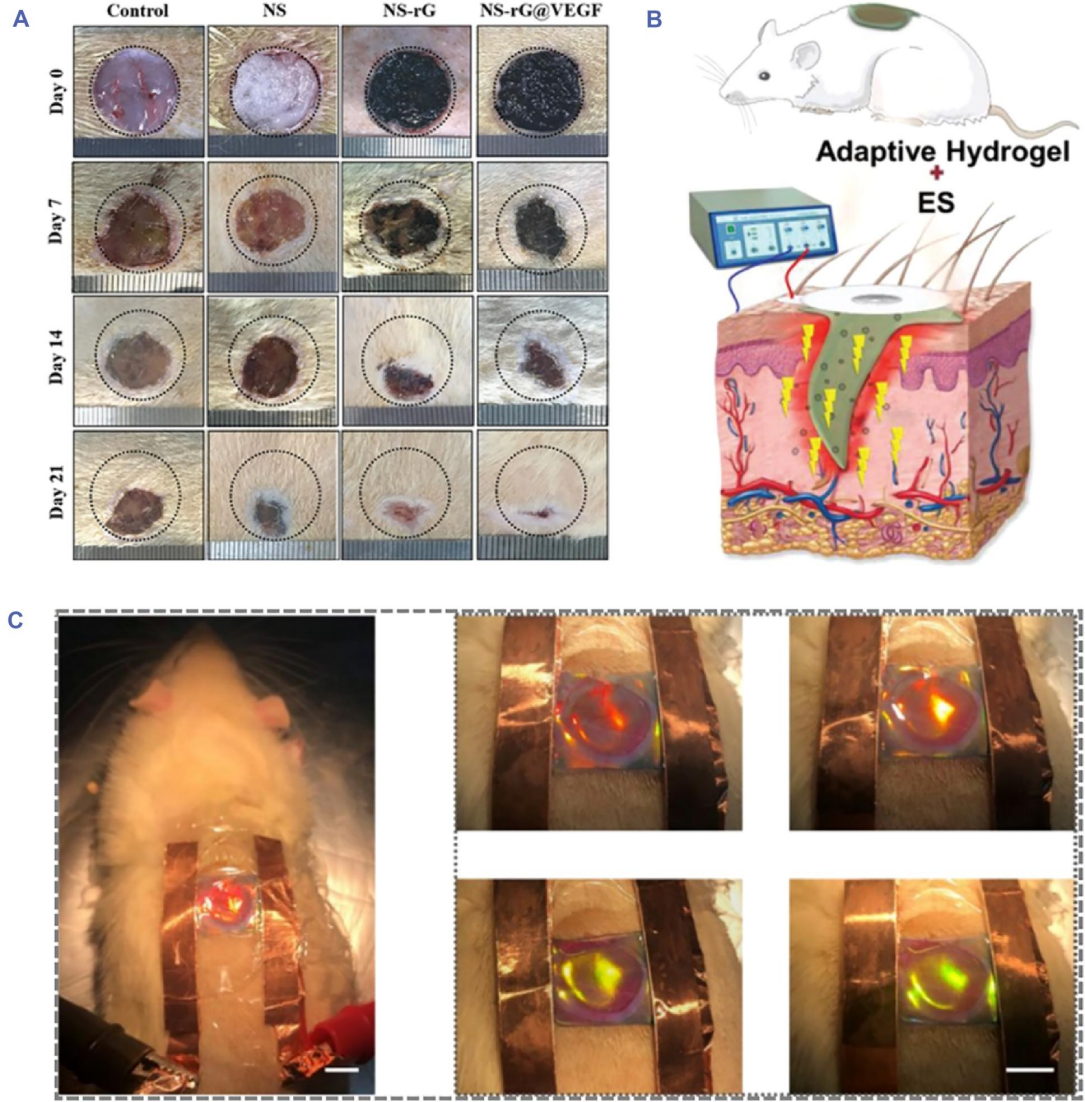
Wound healing applications. (A) NS‐rG@VEGF applied to mice wounds. *Source*: Reproduced with permission.^108^ Copyright 2022, John Wiley and Sons. (B) Schematic of PHTB(TA‐siRNA) using electrical stimulation on the wound tissue. *Source*: Reproduced under terms of the CC‐BY license.^109^ Copyright 2022, The Authors, published by John Wiley and Sons. (C) Images of structural color hydrogel patch adhered on mice wounds with electrical stimulation. *Source*: Reproduced with permission.^93^ Copyright 2023, American Chemical Society.

Benefitting from their capability to transmit electrochemical signals and promote cellular activities, conductive hydrogels have gained extensive interests in the treatment of diseases. In particular, conductive hydrogels have proven to have a positive effect on promoting cell growth, migration and proliferation.^105^, ^106^ As skin is known as the electrical signal‐sensitive tissue, conductive hydrogels have potential for wound treatment. As expected, it has been reported that conductive hydrogels have shown effective therapeutic effects on wound diseases. A PPy‐based collagen conductive hydrogel has been developed for the treatment of full‐thickness wounds.^107^ The presence of PPy imparted the hydrogels with electroactivity, accelerating the wound healing process. Besides, Cui et al. provided a drug‐loaded rGO conductive hydrogel short nanofiber (NS‐rG@VEGF) to explore the effect of endogenous electric fields on the repair of damaged tissue.^108^ It was reported that endogenous electric fields contributed to the generation of bioelectric signals through absorbing wound exudates, which resulted in promoting the diabetic wound healing (Figure 6A).

Apart from the electroactivity nature, conductive hydrogels applied for wound sites under appropriate electrical stimulation have been confirmed to enhance drug release capability, further beneficial for wound healing.^110^ Taking advantage of self‐assembled TA‐short interfering RNA (siRNA) nanogels (TA‐siRNA), Fan's group constructed a conductive nanogels (PHTB(TA‐siRNA)), and investigated the influence of electrical stimulation on the release of TA‐siRNA (Figure 6B).^109^ It was illustrated that the electrical stimulation provoked the release of TA‐siRNA, which was conducive to reduce ROS level; meanwhile, the inflammatory was suppressed by TA. The synergistic effect of electric fields and hydrogels accelerated the healing of defected skin. Despite the realization of controlled drug release by electrical stimulation, the degree of drug release can hardly be evaluated. Recently, Zhao's group proposed a structural color ionic hydrogel patch, which consisted of an inverse opal structured ionic hydrogel layer and a drug‐loaded methacrylated gelatin (GelMA) layer, possessing dual‐signal sensing properties.^93^ Benefitting from the features of color‐sensing and good electrical performance, the drug release process could be visualized and monitored. It was demonstrated that when applied with the electrical stimulation, the drugs were forced to release and the structural color showed a shift from red to orange green, indicating the successful drug release condition (Figure 6C). The effective drug release induced by electrical stimulation was conductive to improve the treatment of diabetic wounds.

### 4.3 Tissue engineering

As introduced that bioelectricity or electrical stimulation has a positive correlation with the tissue growth and behaviors, it is worth noting that such a beneficial effect also favors the tissue regeneration. Specifically, conductive hydrogels with great conductivity can provide a conductive bridge to connect with cells or tissues, promoting their growth and regeneration.^111^ Generally, conductive hydrogels are widely used for the regeneration of neural and cardiac tissues.

**FIGURE 7:**
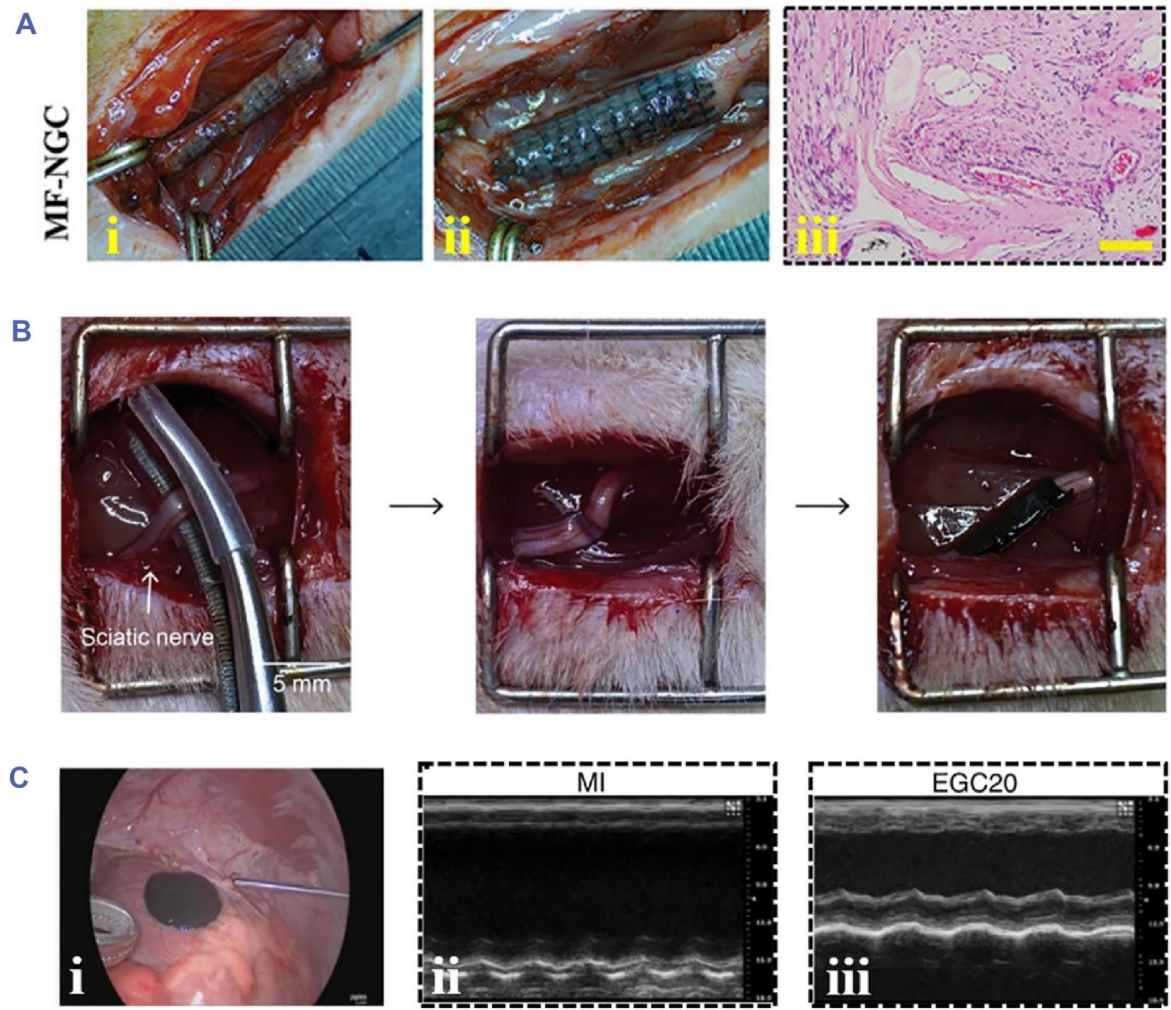
Tissue engineering applications. (A) (i‐ii) Photos of MF‐NGC for peripheral nerve regeneration. (iii) H&E staining. *Source*: Reproduced under terms of the CC‐BY license.^115^ Copyright 2023, The Authors, published by John Wiley and Sons. (B) Images of the conductive composite conduit for sciatic nerve crush model. *Source*: Reproduced with permission.^116^ Copyright 2023, John Wiley and Sons. (C) (i) In vivo injection delivery of EGC20 scaffold on porcine heart. (ii‐iii) Echocardiograms of left ventricular contraction of MI group (ii) and EGC group (iii). *Source*: Reproduced with permission.^117^ Copyright 2021, The Authors, published by Springer Nature.

Peripheral nerve injuries (PNS) have been a common but serious clinical disease, affecting the motor activity and threatening human health. Peripheral nerve regeneration has aroused researchers' attention, and various approaches have been proposed, among which conductive hydrogels are regarded as a promising material to promote nerve tissue regeneration. Two ways based on conductive hydrogels are reported to be useful, involving the incorporation of aligned topological cues and the employment of appropriate electrical stimulation.^112^, ^113^ Specifically, due to the existence of directional axons in nerve cells, the directional arrangement structure of conductive hydrogels is conducive to promoting the growth of directional axons in nerve cells. Nerve guidance conduits (NGCs), which are composed of aligned nanofibers, have been developed for connecting damaged areas and enhancing nerve regeneration.^114^ It was reported that a conductive multiscale NGC (MF‐NGC) had a positive effect on neurite outgrowth of neuronal cells, and hierarchically arranged fibers promoted the regeneration of peripheral nerve tissues (Figure 7A).^115^ In contrast, electrical stimulation has demonstrated to enhance cell proliferation and growth. Jiang et al. fabricated a conductive composite conduit with alignment structure for nerve regeneration.^116^ It was shown from an in vivo rat sciatic nerve crush experiment that when applied with electrical stimulation, the myelin sheath was induced to grow and nerve tissues were promoted to regenerate with a faster recovery rate (Figure 7B), indicating the remarkable value of electrical stimulation on nerve regeneration.

Cardiovascular disease, especially myocardial infarction (MI), is one of the most deadly diseases worldwide. The typical symptoms of MI are asynchronous contraction and arrhythmia. Cardiac patches derived from conductive hydrogels show great therapeutic efficacy against MI by transmitting electrical signals to restore the functions of cardiac tissues involving synchronous contractions and blood supply reconstruction.^118^ However, there remain challenges in delivering conductive patches to cardiac. It was reported that injectable conductive hydrogels could resolve this issue by injecting a prefabricated gel solution into the heart. For instance, Qiu et al. provided an injectable conductive cardiac patch (EGC scaffold) for the treatment of MI.^117^ In order to show the advantage of injectable hydrogel applied in *in vivo* experiment, EGC scaffold was injected on the heart of a porcine model (Figure 7C). It was found that such EGC scaffolds could be successfully delivered and fixed on the porcine’s heart, proving the feasibility of injectable hydrogels. Notably, the cardiac functions of porcine with MI were restored after the delivery of the injectable conductive hydrogels, which could be found from echocardiograms that an evident contraction wave was observed from the EGC group compared to the control group. To overcome the mechanical brittleness and better adapt to the dynamic physiological environment, Qian's group fabricated an injectable conductive hydrogel with self‐healing performance, which made the patch match well with the cardiac tissues, and an improved cardiac function of rats was realized.^119^

## 5 SUMMARY AND OUTLOOK

The past decades have witnessed the advancements of conductive hydrogels. Two types of conductive hydrogels have emerged, including electronic and ionic conductive hydrogels. In this review, we have summarized the latest progress of conductive hydrogels. Especially, to meet the requirements of biomedical fields, conductive hydrogels are endowed with advanced functionalities such as toughness, strong adhesion, self‐healing and color‐sensing. These functionalities have significantly improved the performance of conductive hydrogels and made them exhibit promising prospects in biomedical applications including bioelectronics, wound treatment and tissue engineering.

Despite the thriving scientific progress on conductive hydrogels, some challenges remain and need to be concerned and addressed. The first issue involves the reliability and stability of conductive hydrogels. As hydrogels are composed of hydrophilic 3D porous networks, dehydration is negligible, which may dramatically weaken their mechanical and conductivity properties. It is urgent to seek effective approaches to overcome this issue. Many binary solvents or inorganic salts like glycerin, lithium chloride, lithium chloride and so on can be introduced for building anti‐dehydration conductive hydrogels, which are capable to adverse external environments and show superior stability for sensing. Although with these merits, the types of such hydrogels are still limited, and the exploration of the fundamental mechanism of gelling is a challenge. It is expected to gain insight into the formation mechanism of hydrogels and develop high‐performance conductive hydrogels to improve their reliability.

The second issue refers to the biosafety. Subjected to the material compositions and conductive principles, most conductive hydrogels suffer from poor biocompatibility and biodegradability, which greatly hinder their biomedical applications. For example, for implanted bioelectronics, long‐term in vivo treatments have a huge impact on ionic conductive hydrogels, which will induce the leakage of salts or electrolytes. This situation not only destroys their conductivity but also is harmful to human organs. Besides, the reaction of foreign bodies cannot be ignored. Thus, truly biocompatibility conductive hydrogels are required, and endeavor attempts should be concentrated on the development of bioabsorbable or effective biodegradable materials. The extra introduction of biodegradable materials into the conductive hydrogel systems will be a promising direction, which is conducive to avoiding unnecessary postoperative harm and contributing to further clinical applications.

The third issue regards the application of electrical stimulation. Although electrical stimulation is demonstrated to be valuable for cellular growth and has a positive bioelectrical effect on tissue engineering, different degrees of electrical stimulation will induce an unpredictable outcome. Meanwhile, since electrical stimulation standardization in terms of intensity, frequency, time, etc. is vague, management for the electrical stimulation standardization is critical, which calls for the systematic study of the effect of electric fields or current on cells or tissues.

In conclusion, we have concentrated on recent progress in advanced functions and biomedical applications of conductive hydrogels. Despite the existing challenges, the advances in material science, manufacture technology, and clinical medicine will promote the advancement of next‐generation conductive hydrogels. We do believe that the future conductive hydrogels will open up new opportunities for clinical applications.

## AUTHOR CONTRIBUTIONS

Yuanjin Zhao provided the idea; Yu Wang wrote the manuscript; Jiahui Guo and Xinyue Cao revised the manuscript.

## CONFLICT OF INTEREST STATEMENT

The authors declare that there are no competing interests.
